# Lysophosphatidylcholine Triggers TLR2- and TLR4-Mediated Signaling Pathways but Counteracts LPS-Induced NO Synthesis in Peritoneal Macrophages by Inhibiting NF-κB Translocation and MAPK/ERK Phosphorylation

**DOI:** 10.1371/journal.pone.0076233

**Published:** 2013-09-30

**Authors:** Alan Brito Carneiro, Bruna Maria Ferreira Iaciura, Lilian Lie Nohara, Carla Duque Lopes, Esteban Mauricio Cordero Veas, Vania Sammartino Mariano, Patricia Torres Bozza, Ulisses Gazos Lopes, Georgia Correa Atella, Igor Correia Almeida, Mário Alberto Cardoso Silva-Neto

**Affiliations:** 1 Instituto de Bioquímica Médica, Programa de Biologia Molecular e Biotecnologia, CCS, Universidade Federal do Rio de Janeiro, Rio de Janeiro, Rio de Janeiro, Brazil; 2 Instituto Nacional de Ciência e Tecnologia em Entomologia Molecular- INCT-EM, Universidade Federal do Rio de Janeiro, Rio de Janeiro, Rio de Janeiro, Brazil; 3 The Border Biomedical Research Center, Department of Biological Sciences, University of Texas at El Paso, El Paso, Texas, United States of America; 4 Departamento de Biologia Celular e Molecular Patogênicos, Faculdade de Medicina de Ribeirão Preto, Universidade de São Paulo, Ribeirão Preto, São Paulo, Brazil; 5 Laboratório de Imunofarmacologia, Instituto Oswaldo Cruz, Fundação Oswaldo Cruz, Rio de Janeiro, Rio de Janeiro, Brazil; 6 Laboratório de Parasitologia Molecular, Instituto de Biofísica Carlos Chagas Filho, CCS, Universidade Federal do Rio de Janeiro, Rio de Janeiro, Rio de Janeiro, Brazil; Instituto de Ciências Biomédicas / Universidade de São Paulo - USP, Brazil

## Abstract

**Background:**

Lysophosphatidylcholine (LPC) is the main phospholipid component of oxidized low-density lipoprotein (oxLDL) and is usually noted as a marker of several human diseases, such as atherosclerosis, cancer and diabetes. Some studies suggest that oxLDL modulates Toll-like receptor (TLR) signaling. However, effector molecules that are present in oxLDL particles and can trigger TLR signaling are not yet clear. LPC was previously described as an attenuator of sepsis and as an immune suppressor. In the present study, we have evaluated the role of LPC as a dual modulator of the TLR-mediated signaling pathway.

**Methodology/Principal Findings:**

HEK 293A cells were transfected with TLR expression constructs and stimulated with LPC molecules with different fatty acid chain lengths and saturation levels. All LPC molecules activated both TLR4 and TLR2-1 signaling, as evaluated by NF-қB activation and IL-8 production. These data were confirmed by Western blot analysis of NF-қB translocation in isolated nuclei of peritoneal murine macrophages. However, LPC counteracted the TLR4 signaling induced by LPS. In this case, NF-қB translocation, nitric oxide (NO) synthesis and the expression of inducible nitric oxide synthase (iNOS) were blocked. Moreover, LPC activated the MAP Kinases p38 and JNK, but not ERK, in murine macrophages. Interestingly, LPC blocked LPS-induced ERK activation in peritoneal macrophages but not in TLR-transfected cells.

**Conclusions/Significance:**

The above results indicate that LPC is a dual-activity ligand molecule. It is able to trigger a classical proinflammatory phenotype by activating TLR4- and TLR2-1-mediated signaling. However, in the presence of classical TLR ligands, LPC counteracts some of the TLR-mediated intracellular responses, ultimately inducing an anti-inflammatory phenotype; LPC may thus play a role in the regulation of cell immune responses and disease progression.

## Introduction

Pathogen infection relies on the ability to avoid Toll-like receptor (TLR)-mediated signaling and thus the triggering of innate immunity. Such signaling pathways involve pathogen-derived molecules or molecules derived from pathogen surroundings [1-4]. Thus far, 11 human TLRs and 13 murine TLRs have been identified, and each TLR appears to recognize a distinct surface molecule derived from a different microorganism, including bacteria, viruses, protozoa and fungi [5]. These receptors are expressed in different cellular compartments. For instance, TLR1, TLR2, TLR4, TLR5, TLR6, and TLR11 (the last of which is expressed only in mice) are expressed on the cell surface, whereas TLR3, TLR7, TLR8, and TLR9 are expressed in intracellular vesicles, such as endosomes and the endoplasmic reticulum (ER). TLRs 1, 2, 4, and 6 recognize lipids and lipid-containing molecules, TLR5 recognizes protein ligands, and TLRs 3, 7, 8, and 9, which are located intracellularly, detect nucleic acids derived from viruses, bacteria, and protozoa [5].

The oxidation of LDL particles is a key event in the development of coronary artery disease and atherosclerosis. In vertebrate plasma, lysophosphatidylcholine (LPC) is a glycerophospholipid produced as a hydrolysis product of phosphatidylcholine (PC) by PAF-acetylhydrolase when in contact with oxidized low-density lipoprotein (oxLDL) [6-8]. LPC in oxLDL is largely involved in the pathogenesis of inflammatory diseases, such as atherosclerosis, psoriasis, asthma, rhinitis, and human lupus erythematous [9-14]. LPC is usually associated with proinflammatory oxLDL particles, and it mimics several intracellular signaling events triggered by the whole oxidized lipoprotein particle. Stewart et al. [15] demonstrated that oxLDL triggers inflammatory signaling through a heterodimer of TLRs 4 and 6. The assembly of this newly identified heterodimer is regulated by signals from the scavenger receptor CD36, a common receptor for these disparate ligands. However, the ligand molecule that is present on oxLDL and is responsible for such events has not been identified.

We have originally demonstrated that LPC is a saliva component in blood-sucking arthropods [16]. LPC blocks the production of the microbicidal molecule nitric oxide (NO), induced by macrophages stimulated with either *Trypanosoma cruzi* or lipopolysaccharide (LPS) [2]. Dyslipidemia inhibits TLR-induced the activation of dendritic cells, thus affecting host resistance to *Leishmania major* [17]. Moreover, it was recently demonstrated that oxLDL inhibits TLR2 and TLR4 cytokine responses in human monocytes [18]. The production of IL-6, IL-1β, TNF, and IL-8 in monocytes was downregulated in the presence of LPS, the monoacylated lipopeptide Pam3CSK4, and oxLDL. Thus, stimulation of TLR2 and TLR4 with classical ligands, such as Pam3CSK4 and LPS, in the presence of oxLDL seems to counteract the inflammatory response usually induced by such ligands. Different pathogens may therefore benefit from a proinflammatory molecule such as LPC during the course of host infection. However, the cellular signaling pathway that mediates these biological effects has not been well studied.

Here, we show that LPC triggers TLR-mediated signaling when presented alone to cells transfected with TLRs. However, when murine peritoneal macrophages are treated with classical TLR ligands in the presence of LPC, LPC counteracts NO production through the reversal of LPS-induced NF-қB translocation to the nucleus and ERK activation. This research suggest that LPC-triggered cascade of events in which the simultaneous stimulation of TLRs leads to the downregulation of the NO synthesis. The present study may reveal an ancient strategy for subversion of TLR-mediated signaling, which allows pathogen infection of vertebrate cells.

## Results

### 1. LPC triggers TLR-mediated signaling in TLR-transfected cells

**Figure 1:**
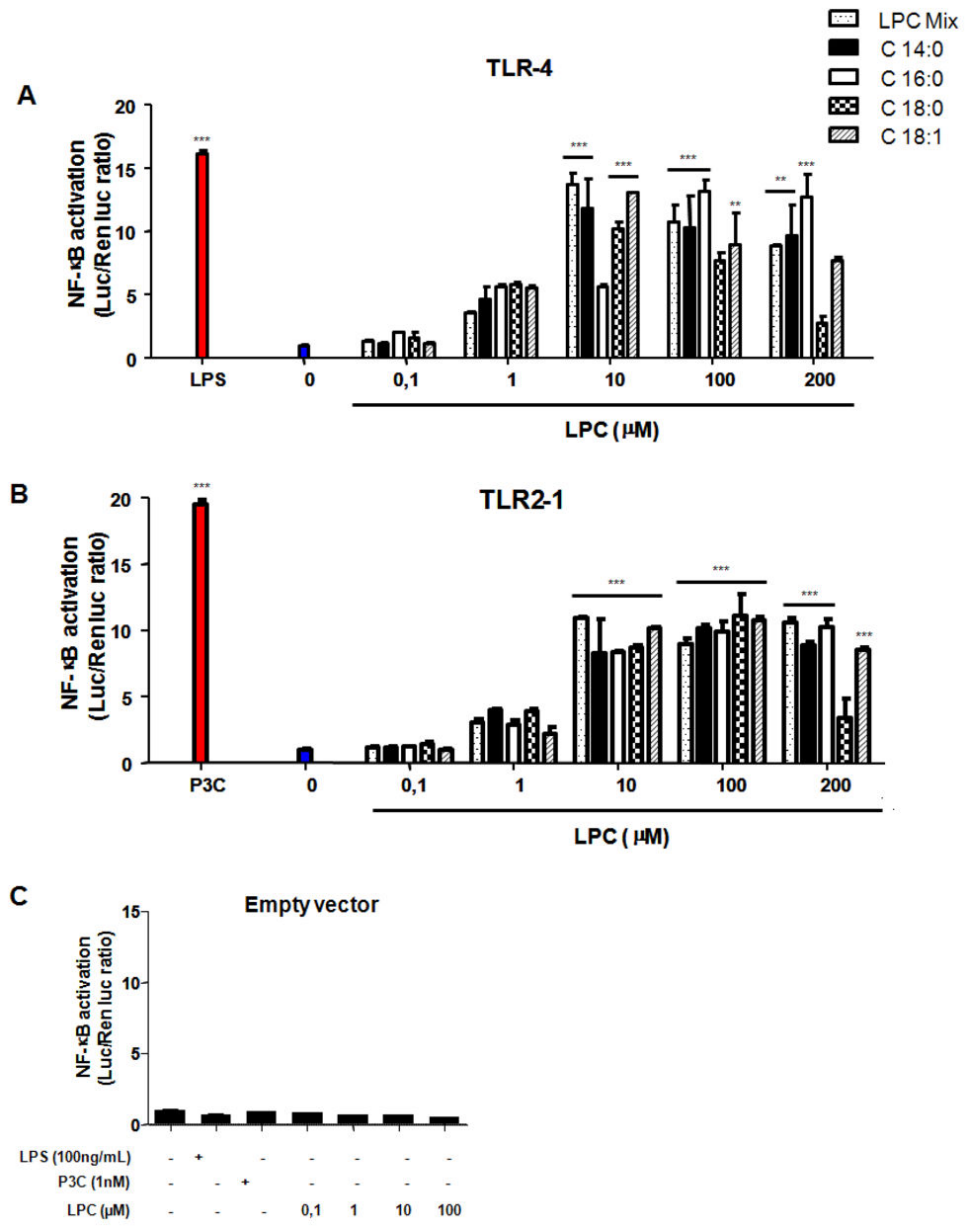
LPC triggers NF-қB activation through either TLR4- or TLR2/1-dependent signaling pathways. HEK 293A cells were transfected in three different groups. Groups A and B received expression constructs for TLR4 (**A**) or TLR2 and TLR1 (**B**). Both also received MD-2, CD14, and CD36 constructs and the ELAM-1-firefly luciferase and β-actin-*Renilla* luciferase reporter plasmids. The third group (**C**) received only the empty vector pDisplay and the luciferase reporter plasmids. Groups A and B were separately stimulated with 0.1, 1, 10, 100 and 200 µM of different types of LPC (Sigma; C14:0, C16:0, C18:0, and C18:1), 100 ng/mL of LPS and 1 nM of Pam3CSK4 (P3C). Group C was stimulated with LPS, Pam3Cys or 0.1, 1, 10 and 100 µM of LPC (C16:0). The agonists were diluted in DMEM medium with 10% bovine fetal serum. After 4 h of incubation, luciferase activity was measured and expressed as the ratio of NF-қB-dependent firefly luciferase activity to the control *Renilla* luciferase activity. Data is the mean ± S.E. of two different experiments. ** P < 0.01, *** P < 0.001 (One way ANOVA, Parameter, Bonferroni’s Multiple Comparison Test).

**Figure 2:**
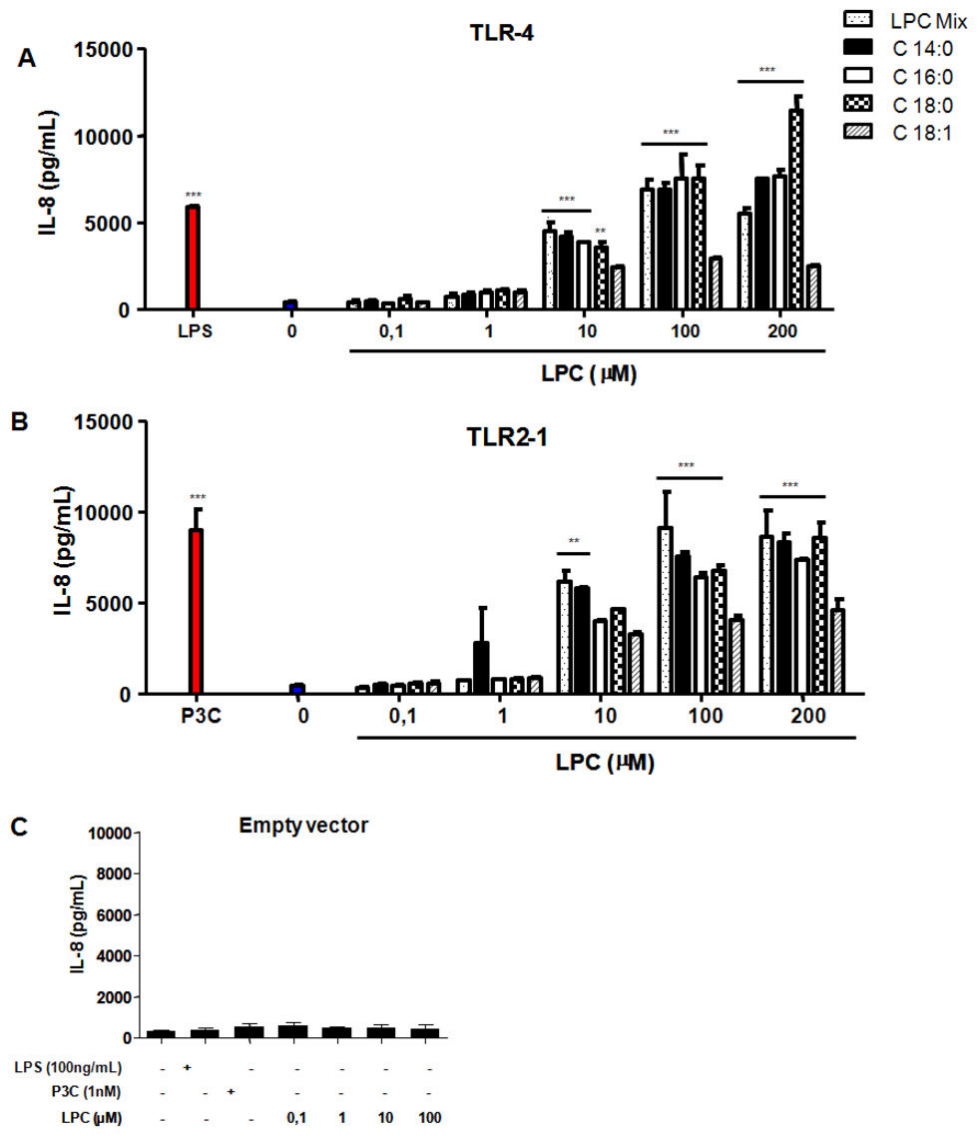
LPC triggers IL-8 production through either TLR4- or TLR2/1-dependent signaling pathways. HEK 293A cells were transfected and stimulated as described on Figure 1. After 20 hours of incubation, IL-8 production was measured by the ELISA assay. Data is the mean ± S.E. of two different experiments. ** P < 0.01, *** P < 0.001 (One way ANOVA, Parameter, Bonferroni’s Multiple Comparison Test).

To clarify the role of LPC in TLR-mediated cell signaling, we transfected HEK 293A cells with constructs expressing TLR4 or both TLR2 and TLR1 (TLR2/1). Treatment of these cells with the classical ligands for TLR4 (LPS) or TLR2/1 (Pam3CSK4 and P3C) induced NF-κB translocation to the nucleus (Figure 1A and 1B). LPCs with different fatty acid chains (i.e., C14:0, C16:0, C18:0, or C18:1) were also able to trigger both NF-κB translocation (Figure 1A and 1B) and IL-8 secretion (Figure 2) in a dose-dependent manner. Cells transfected with the empty vector did not display NF-κB translocation (Figure 1C) or detectable levels of secreted IL-8 (Figure 2C), indicating that such events were dependent on the TLR expression constructs and not mediated by other receptors in HEK 293A cells.

### 2. LPS induction of NO synthesis is blocked by LPC

**Figure 3:**
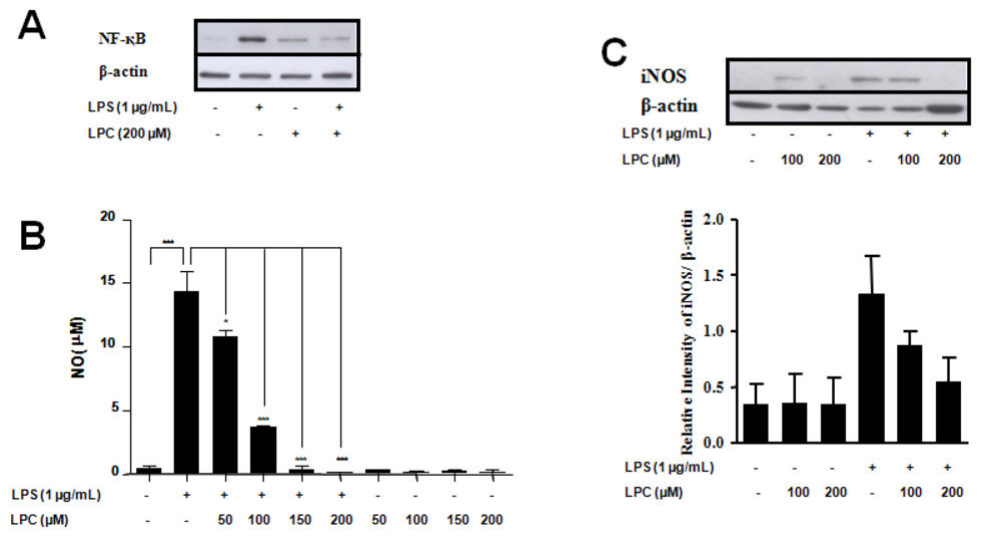
LPC inhibits NF-қB translocation, iNOS expression, and NO production in LPS-stimulated macrophages. Peritoneal macrophages from BALB/cmice were incubated in the absence or presence of 1 µg/mL LPS and different concentrations of LPC (Sigma) at 37 °C in a 5% CO_2_ atmosphere. After 1 h of incubation, NF-қB translocation (**A**) was assayed by Western blot analysis. After 24 hours, NO production (**B**) was assayed by measuring the amount of nitrite in the culture supernatant using the Griess reagent, and iNOS expression (**C**) was determined by Western blot analysis followed by densitometry (lower panel). Data is the mean ± S.D. of three different experiments. * P < 0.05, ** P < 0.01, *** P < 0.001 (One way ANOVA, Parameter, Bonferroni’s Multiple Comparison Test).

We have previously shown that LPC blocks NO production by peritoneal macrophages that are induced with *T. cruzi*, IFN-γ, LPS or LPS/IFN-γ [2]. Treatment of macrophages with LPS classically induces NO synthesis by activating the TLR4-dependent pathway. As shown above, LPC is a ligand able to trigger both TLR1/2- and TLR4-mediated signaling pathways. However, the mechanism by which LPC modulates TLR-mediated signaling is unknown. We next evaluated the effect of LPC treatment on peritoneal macrophages in the presence or absence of LPS. The treatment of cells with either LPS or LPC induced detectable levels of NF-κB in the cell nuclei (Figure 3A), confirming that both ligands trigger a proinflammatory phenotype, as shown above. However, treatment of macrophages simultaneously with both ligands largely decreased the levels of NF-қB relative to those detected with LPS alone (Figure 3A). This result indicates that in the presence of both ligands, TLR-mediated signaling is considerably reduced. LPC activation of TLRs may be affected by the presence of different neighboring receptors or modulated by a crosstalk between intracellular routes. To test this possibility, we followed NO synthesis in LPC-treated cells. The treatment of cells with LPC did not induce NO production, and when added in the presence of LPS, LPC blocked NO synthesis (Figure 3B). The expression of nitric oxide synthase (iNOS) was detected in both LPC- and LPS-treated cells, but incubation of macrophages largely decreased iNOS levels (Figure 3C). At 200 μM LPC (Figure 3C), the levels of both NF-κB and NO were negligible (Figure 3A and 3B).

### 3. LPC blocks LPS-induced ERK activation in peritoneal macrophages but not in TLR-transfected cells

**Figure 4:**
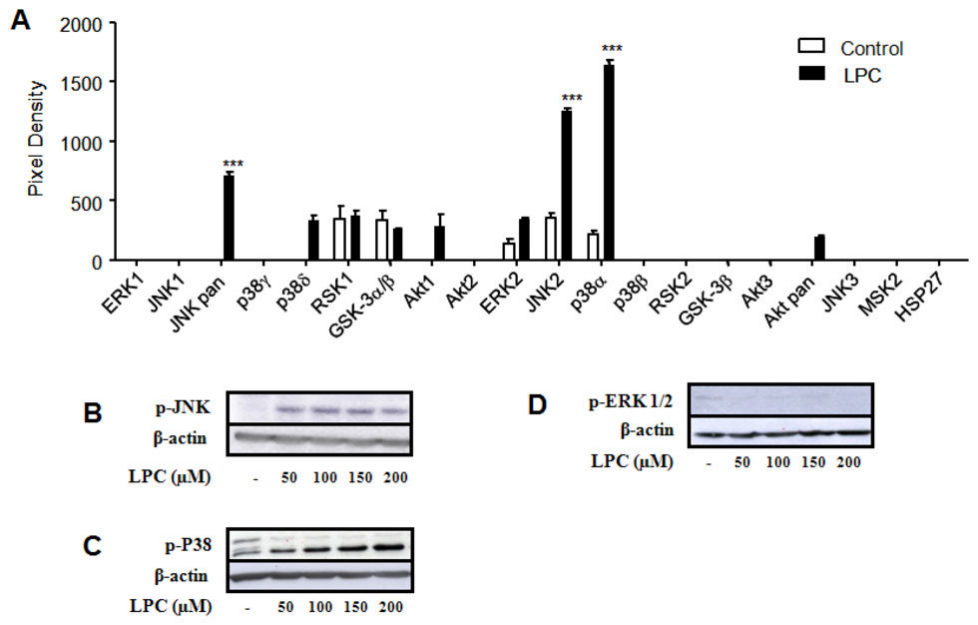
LPC activates JNK and p38, but not ERK, in macrophages. Peritoneal macrophages from BALB/c mice were incubated in the absence or presence of different concentrations of LPC mix (Sigma) for 20 min at 37 °C in a 5% CO_2_ atmosphere, and the cytoplasm content was homogenized and assayed as follows. The Phospho-MAPK array was used for analysis of enzymatic activation (**A**). The reaction was visualized with the enhanced chemiluminescent system and subjected to densitometric analysis (***, p< 0.001, ANOVA). Protein levels of the phosphorylated MAPKs JNK (**B**), p38 (**C**) and ERK (**D**) were determined by Western blot. Data is the mean ± S.E. of two different experiments.

**Figure 5:**
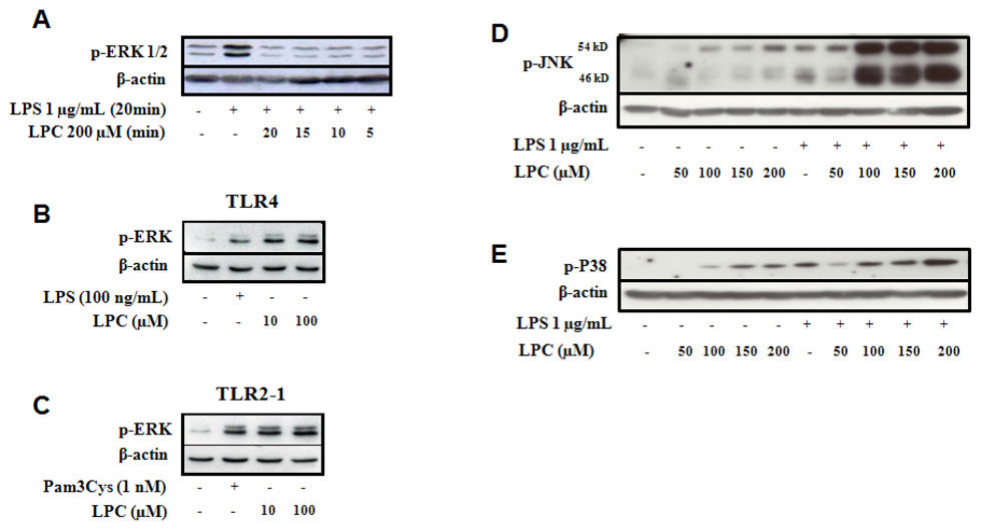
LPC inhibits LPS-induced ERK activation. Peritoneal macrophages from BALB/c mice were incubated in the absence or presence of 1 µg/mL LPS or in the presence or absence of the indicated concentrations of LPC (Sigma) at 37 °C in a 5% CO_2_ atmosphere (**A**, **D**, **E**). In parallel HEK 293A cells with TLR constructs as indicated (**B**, **C**). Each group received expression constructs for TLR4 (**B**) or both TLR2 and TLR1 (**C**), as well as MD-2, CD14 and CD36 plasmids. The cells were then incubated in the absence or presence of 100 ng/mL LPS or 1 nM Pam3CSK4 (P3C) and 10 or 100 µM of LPC, for 40 min at 37 °C in a 5% CO_2_ atmosphere. After incubation either macrophages or HEK cells were homogenized, the protein levels was determined and samples evaluated by Western blot with the use of antibodies against p-ERK (**A**, **B**, **C**), p-JNK (**D**) and p-P38 (**E**). Loading controls were run with the use of antibodies raised towards actin. Experiments were performed at least two times with different animals and samples.

An important step in TLR-mediated signaling is the downstream activation of mitogen-activated protein kinases (MAPKs). LPC was able to induce phosphorylation of the MAPKs JNK and p38, but not ERK, as assayed by the Phospho-MAPK Array (Figure 4) and by western blotting for each MAPK subfamily. We next compared the dynamics of ERK phosphorylation in peritoneal macrophages and TLR-transfected HEK cells. ERK activation in peritoneal macrophages induced by LPS was blocked by incubation with LPC (Figure 5A). However, the treatment of TLR-transfected cells with either LPS or Pam3CSK4 induced ERK phosphorylation (Figure 5B and 5C). Incubation of cells with both LPS and LPC did not counteract LPS-induced activation of JNK and p38 (Figure 5D and 5E). These results show that LPC acts as proinflammatory molecule able to trigger TLR-mediated signaling when TLRs are expressed in HEK cells. However, the treatment of macrophages with presence LPC and LPS a classical TLR ligand leads to a counteraction of LPS-induced ERK activation but not of JNK and p38. Thus LPC prevents the triggering of an effective and complex innate immune response and is somewhat able to block the induction of a proinflammatory phenotype.

## Discussion

Receptors involved in phospholipid signaling often display significant promiscuity, recognizing several different ligands. Different receptors have been proposed for LPC, including G2A and GPR4 [19,20], which are both coupled to G proteins. However, the original studies describing G2A and GPR4 as LPC receptors were based on binding assays using radiolabelled lysophospholipids and are thus difficult to reproduce by other groups. The role of G2A and GPR4 as high-affinity receptors for LPC thus remains controversial. In the present study, we have shown that LPC is an activator of TLR2/1- and TLR4-mediated signaling pathways (Figures 1 and 2). Except for C18:0-LPC, differences in fatty acid chain length or saturation in the *sn*-1 position of the glycerol moiety of LPC had no major effect on TLR activation. When added to TLR4-transfected HEK 293A cells, C18:0-LPC generated a profile of NF-қB activation that did not match the levels of IL-8 production (Figures 1 and 2). This result suggests that under such conditions, signaling pathways independent of NF-қB are being activated. To verify that this LPC effect was occurring via TLRs, we transfected HEK 293A cells with the empty vector and showed that the activation of NF-қB and the production of IL-8 were totally abolished (Figures 1C and 2C). These data align with previous studies that used indirect approaches to demonstrate TLR activation through the action of oxidized LDL [21-24]. Moreover, regarding pathogen-host interactions, it was previously shown that LPC derived from *Schistosoma mansoni* induces the production of inflammatory cytokines in murine macrophages by a mechanism dependent on TLR2 [25].

The molecular mechanisms regulating iNOS transcription have been well studied in different cell types. In macrophages, for example, the enzyme is primarily regulated by cellular receptors, such as TLRs, and their accessory molecules. The promoter region of the iNOS gene contains several binding sites for transcription factors, including NF-қB, AP-1, Jun / Fos, the CREB family, and STATs [26-29]. However, induction of iNOS expression by LPS signaling occurs through TLR4, which activates only NF-қB and AP-1 [28,29]. The translocation of NF-қB is significantly reduced in murine macrophages stimulated with LPS in the presence of LPC. Therefore, we suggest that the inhibition of NO production by LPC results from lower iNOS expression levels, which is due to the reduced translocation of NF-қB. Interestingly, LPC was also able to activate NF-қB in different cell types when incubated in the absence of LPS, as shown by two different assays (Figures 1-3). These results demonstrate that when added separately, both LPS and LPC might be signaling through TLR4. This hypothesis is also supported by studies of TLR expression in atherosclerotic lesions, which suggest that TLR signaling and the NF-қB pathway are activated by the presence of oxLDL containing LPC on its surface [23,30]. Thus, LPC from oxLDL is a major regulatory molecule that activates a variety of genes involved in atherosclerosis and the inflammatory response; this effect leads to cytokine release and eventually impacts the adaptive immunity. The present study supports this idea through assays using cells transfected with different TLRs receptors.

LPC has been identified as a candidate therapeutic molecule for treatment of sepsis [31]. Septicemia is a violent response of the body to an infection; with no specific therapy available, it is currently the leading cause of death in intensive care units in hospitals. The experimental data suggest that LPC can efficiently reduce the degree of injury and systemic dysfunction caused by sepsis because it suppresses the inflammatory responses of the host; LPC may thus be an effective agent for the prevention and treatment of this disease [32,33]. A mechanism was recently proposed, by which LPC blocks TLR4 translocation to lipid raft microdomains in the membrane [34].

MAPKs are involved in the regulation of inflammation mediators such as NO. Thus, we have examined p38, JNK, and ERK activation by LPC in peritoneal macrophages. Two different experimental approaches demonstrated that LPC activated JNK and p38 but not ERK (Figure 4 and Figure 5). Furthermore, LPC was able to block LPS-induced ERK activation (Figure 5A). However, unlike its action in peritoneal macrophages, LPC activated ERK in HEK 293A cells transfected with TLR4 or TLR2-1, as shown in Figure 5D and 5E. These results may be due to the fact that HEK 293A cells are devoid of many receptors, and possibly a specific receptor for LPC. Macrophages, on the other hand, as cells engaged in innate immunity, display a wider range of membrane receptors. The inhibition of ERK by LPC in LPS-stimulated murine macrophages could thus occur through one of such receptors, which triggers the activation of downstream MAPK phosphatases (MKPs); the MPKs may then negatively regulate ERK kinase phosphorylation [35]. This hypothesis is supported by a recent report, which shows that activation of MAPK phosphatases protects the organism from sepsis-induced acute lung injury [36].

The findings of the present work have some major implications to the biology of TLRs and innate immunity. It has long been recognized that such receptors display synergy and cross-tolerance [37]. Thus LPS exposed cells down regulate the surface exposition of TLR4 receptors which led to hyporesponsiveness to the following LPS expositions. The mechanism of LPC-mediated suppression of LPS-induced NO production as demonstrated in the present study may share some common events to the classically described cross-tolerance of TLRs. Regarding this subject once LPS activates several downstream transcriptional factors through MAPK-mediated pathways such as SRE, AP-1, CRE and NF-қB [38]. In the present study we have demonstrate a partial impairment of NF-қB translocation to the nucleus but the involvement of further transcriptional factors was not evaluated. This is especially important regarding the activation of p38 and JNK in the presence of LPC. TLRs are associated primarily with immune cells, however they are also present on the surface of other cell types such as renal epithelial cells. It is reported that previous exposition to such cells to oxalate implies on a loss of responsiveness to LPS. Also prior exposure to LPS triggers a similar desensitization to oxalate stimulation. It is curious that oxalate exposure activates PLA_2_ which then generates LPC inside renal cells. So oxalate-induced hyporesponsiveness to LPS is likely mediate by LPC in this model [39]. Regarding the involvement of LPC in oxLDL-mediated chronic inflammation our current findings demonstrate by an independent approach the role of LPC in this pathological process. These data are supported by previous demonstrations that oxLDL uncouples TLR-mediated signaling on dendritic cells, inhibits NF-κB nuclear translocation and suppress LPS or PamCSK4 induced synthesis of cytokines such as IL-1β, TNF and IL-6 [17,18]. oxLDLs but not native LDL also counteract the production of the anti-inflammatory cytokine IL-10 production by monocytes responding to TLR2 and TLR4 [40]. Curiously, oxLDL also suppress the production of IL-10 by monocytes challenged with LPS derived from some orally available bacteria [40]. Another aspect that must be emphasized is the role played by oxLDL in the regulation of iNOS expression. Huang et al. 1999 [41] demonstrated that oxLDL but not LDL blocks NO production through the inhibition of iNOS expression in LPS-stimulated macrophages. Also, Schmid et al. 2008 [42] demonstrated that hypochlorite-oxidized LDL also decreases iNOS and NO production in macrophages. Such studies provided evidence that oxLDL and probably LPC modulate several aspects of NOS biology, including gene transcription, and translation. In summary, these later results combined with our previous studies on the role of LPC in *T. cruzi* infection [2,16,43] suggest that the handling of innate immunity either by invading pathogens or during the natural course of inflammatory process may be conducted by available lysophospholipids.

## Materials and Methods

### Experimental Animals

All animal care and experimental protocols were conducted following the guidelines of the institutional care and use committee (Committee for Evaluation of Animal Use for Research from the Federal University of Rio de Janeiro, CAUAP-UFRJ) and the NIH Guide for the Care and Use of Laboratory Animals (ISBN 0-309-05377-3). The protocols were approved by CAUAP-UFRJ under registries #IBQM027. Technicians dedicated to the animal facility at the Institute of Medical Biochemistry (UFRJ) carried out all aspects related to mice husbandry under strict guidelines to insure careful and consistent handling of the animals. Animals were BALB/c 4-6 week old male mice.

### Cell Populations

Murine peritoneal macrophages from BALB/c mice were obtained 4 days after the injection of 4% thioglycolate. 2-5 x 10^5^ cells were plated in 96-well culture plates (NO assays) or 2 x 10^6^ cells were plated in 6-well culture plates (Western blot analysis) and were cultured in RPMI 1640 medium (Invitrogen, Grand Island, NY) supplemented with 100 U/mL penicillin and 100 µg/ml streptomycin, at 37 °C under 5% CO_2_ atmosphere. HEK 293A (human embryonic kidney, adherent clone) cells were cultured in high-glucose Dulbecco’s modified Eagle medium (DMEM) supplemented with 10% fetal bovine serum (FBS) in the absence of antibiotics, at 37 °C under 5% CO_2_ atmosphere.

### Analysis of NF-kB Translocation in Peritoneal Macrophages

After the indicated incubation, wells containing cells were washed twice with PBS and the cells were transferred to eppendorfs tubes. The cells were centrifuged for 10 min, 1500 rpm, resuspended in 450 µL of cold buffer A (10 mM HEPES, 10 mM KCl, 100 µM EDTA, 100 µM EGTA, inhibitor protease cocktail), incubated for 15 min and then 50 µL of 5% NP-40 were added to each sample. Samples were then centrifuged for 7 min at 4 °C for 6000 rpm. The supernatant was discarded and the pellet was washed once with 250 µL of cold buffer A. Then, they were centrifuged for 7 min at 4 °C, 9000 rpm, the pellet was resuspended with 50 µL of buffer C (20 mM HEPES, 0.4 M NaCl, 1 mM EDTA, 10 mM EGTA, 1 mM DTT, inhibitor protease cocktail) and incubated for 30 min on ice. Finally samples were then centrifuged for 10 min at 4 °C, 12000 g, the supernatants were collected and analyzed by western blot. We used β-actin as loading control [44].

### Plasmid Constructs

The mouse CD14, MD-2, and HA epitope-tagged Toll-like receptor (TLR) constructs, as well as the β-actin-*Renilla* luciferase and the ELAM-1-firefly luciferase reporter constructs, were kindly provided by Dr. Richard Darveau (University of Washington, Seattle, WA). The mouse CD36 construct was generously provided by Dr. Kathryn J. Moore (Harvard Medical School, Boston, MA). Mouse CD14 was cloned into the expression vector pcDNA3.1/Zeo (Invitrogen) [45]. Mouse MD-2 was cloned into the pEFBOS vector [46,47]. Mouse TLR1, TLR4, and TLR6 were cloned into a modified pDisplay vector (Invitrogen), which provides an ER signal peptide and an amino-terminal hemagglutinin (HA) tag. In the modified pDisplay vector, the c-Myc epitope tag and the PDGFR transmembrane domain have been deleted [48]. Mouse TLR2 was cloned into a modified pDisplay vector (Invitrogen) in which the neomycin-resistance cassette had been replaced by a Zeocin-resistance cassette [45]. To generate the ELAM-1-firefly luciferase construct, the E-selectin promoter, containing an NF-κB binding site, was cloned upstream of the firefly luciferase reporter gene in the pGL2-basic plasmid (Promega) [49]. The β-actin-*Renilla* luciferase construct was generated by cloning the β-actin promoter upstream of the *Renilla* luciferase gene in the pRL-null plasmid (Promega) [50]. Mouse CD36 was cloned into pcDNA3.1/hygro (Invitrogen) [51]. Essentially, in this system luciferase activity is given by the ratio of NF-κB-dependent firefly luciferase activity, an enzyme used in oxidative chemiluminescence and only expressed when its gene undergoes transcription mediated by NF-қB, to *Renilla* luciferase activity, a protein constitutively expressed.

### TLR Transfection Assays with HEK 293A Cells

For transfection assays, HEK 293A cells were plated in 12-well plates (Invitrogen) at 5 x 10^5^ cells per well and transfected after 24 h using Lipofectamine 2000 and Opti-MEM (Invitrogen), according to the manufacturer’s instructions. Amounts of construct per well were 0.22 µg mouse TLR2 or TLR4, 0.22 µg mouse MD-2, CD14, or CD36, 0.88 µg mouse TLR1, 0.22 µg firefly luciferase reporter construct driven by an NF-қB-dependent promoter (ELAM), and 7.4 ng of the *Renilla* luciferase reporter construct (β-actin-*Renilla* luciferase, used to control for transfection efficiency). Cells were grown for 24 h following transfection, detached from the plastic by mild trypsin treatment, and plated on 96-well plates at 4 x 10^4^ cells per well. After 24 h, cells were stimulated for 4 h for the luciferase reporter assay or for 20 h for IL-8 detection, at 37 °C under 5% CO_2_ atmosphere. All stimuli were diluted in DMEM containing 10% FBS.

### Luciferase Reporter Assays for NF-κB Activation

Transfected HEK 293A cells were treated with the following stimuli: 100 ng/mL of LPS (LPS-E Ultrapure, Invivogen, San Diego, CA); 1 nM of Pam3CSK4 (Invivogen, San Diego, CA); 0.1, 1, 10, 100 or 200 µM of LPC mix (L4129, Sigma-Aldrich, St. Louis, MO) or LPC molecules with different fatty acid chain length or saturation including C14:0-, C16:0-, C18:0- and C18:1- (Avanti Polar Lipids, Alabama, USA). After 4-h of stimulation, transfected HEK 293A cells were washed once in PBS and lysed in Passive Lysis Buffer (Promega, Madison, WI) according to the manufacturer’s instructions. The luciferase activity was measured using the Dual-Luciferase Reporter Assay System (Promega) according to the manufacturer’s instructions. The relative luminescence units (RLU) were quantitated using the Luminoskan Ascent luminometer (Thermo Scientific). The values of the non-stimulated cells for each transfected cells were subtracted from the respective stimulated transfected cells. Luciferase activity was expressed as the ratio of NF-κB-dependent firefly luciferase activity to constitutively expressed *Renilla* luciferase activity.

### IL-8 Quantification

Transfected HEK 293A cells were stimulated as described above. After 20 h, cell culture supernatants were collected, and IL-8 levels were determined using the OptEIA Human IL-8 ELISA Kit (BD Biosciences, San Jose, CA) according to the manufacturer’s instructions, except that the Super Signal chemiluminescent reagent (Pierce, Thermo Scientific, Rockford, IL) was used for detection; the RLUs were measured in a Luminoskan Ascent (Thermo Scientific).

### Nitric Oxide Quantification

Murine peritoneal macrophages as platted in 96-wells culture plates, obtained as described above, were stimulated with LPS (1 µg/ml) in the presence or absence of different concentrations of LPC (L4129, Sigma-Aldrich, St. Louis, MO) and 37.5 µM fatty acid-free bovine serum albumin (A7030, Sigma-Aldrich, St. Louis, MO). Twenty-four hours later, the level of nitric oxide, as measured by nitrite production, was determined using the Griess reagent (Invitrogen).

### MAP Kinase Array Analysis of BALB/c Peritoneal Macrophages

Peritoneal macrophages from BALB/c mice were collected, placed onto six-well culture plates (Invitrogen) and left to adhere for 20 min at room temperature. Cells were then incubated with 1 mL RPMI 1640 medium in the absence or presence of 200 µM LPC (L4129, Sigma-Aldrich, St. Louis, MO) and 37.5 µM fatty acid-free BSA for 20 min at 37°C, under 5% CO_2_ atmosphere. Cells were homogenized in PBS pH 7.4, 1 mM EDTA, 1 mM EGTA, 0.15 mM OKA (okadaic acid), 0.18 mM PAO (phenylarsine), and a cocktail of protease inhibitors (1.04 mM AEBSF, 0.8 μM aprotinin, 20 μM leupeptin, 40 μM bestatin, 15 μM pepstatin A, and 14 μM E-64) from Sigma-Aldrich (St. Louis, MO). The homogenates were assayed using the Human Phospho-MAPK Array (R&D Systems Inc., Minneapolis, MN). The reaction was visualized with the enhanced chemiluminescent system and was then subjected to densitometric analysis by the Image Total Lab program (Version 1.11, Phoretix, NE, UK).

### SDS-PAGE and Western Blotting

Following incubation of 2 x 10^6^ peritoneal macrophages from BALB/c mice, cells were washed with 0.9% saline and homogenized with 10 mM Tris-HCl buffer (pH 7.4), 150 mM NaCl, 0.5% NP-40, 10% glycerol (v/v), 1 mM DTT, 0.1 mM EDTA, 1 mM sodium vanadate, 25 mM NaF, 1 mM PMSF, and a cocktail of protease inhibitors (1.04 mM AEBSF, 0.8 μM aprotinin, 20 μM leupeptin, 40 μM bestatin, 15 μM pepstatin A, and 14 μM E-64) from Sigma-Aldrich (St. Louis, MO) [52]. Proteins from cell homogenates (30-50 μg) were separated by running on 10% SDS-polyacrylamide gels at a constant current of 16 mA. The Spectra™ Multicolor Broad Range Protein Ladder (Fermentas Molecular Biology Tools, Thermo, Fisher Scientific) was used as a relative molecular mass standard. After the gel run, the samples were transferred to a PVDF membrane in a buffer of 25 mM Tris–HCl pH 8.3 and 192 mM glycine, at 190 mA for 90 min at 4 °C. The membranes were blocked with a TBS solution containing 0.1% Tween-20 and 5% BSA for 1 h and then incubated for 18 h with monoclonal antibodies (1:1000) raised against phospho-ERK, phospho-JNK, or phospho-p38 (sc-7383, sc-6254 and sc-7973, respectively, from Santa Cruz Biotechnology, CA). The membranes were then incubated with a secondary antibody (anti-mouse IgG-HRP, Santa Cruz Biotechnology, CA), followed by detection with the ECL chemiluminescent reagent (GE Healthcare Life Sciences, Piscataway, NJ).

### Statistical Analysis

The results are presented as the mean and standard error of the mean SE. Normalized data were analyzed by one-way analysis of variance (ANOVA) followed by Bonferroni’s Multiple Comparison Test using the software GraphPad Prism.

## References

[1] Olivier M, Gregory DJ, Forget G (2005) Subversion mechanisms by which Leishmania parasites can escape the host immune response: a signaling point of view. Clin Microbiol Rev 18: 293-305. doi:10.1128/CMR.18.2.293-305.2005. PubMed: 15831826. 15831826 PMC1082797

[2] Mesquita RD, Carneiro AB, Bafica A, Gazos-Lopes F, Takiya CM et al. (2008) Trypanosoma cruzi infection is enhanced by vector saliva through immunosuppressant mechanisms mediated by lysophosphatidylcholine. Infect Immun 76: 5543-5552. doi:10.1128/IAI.00683-08. PubMed: 18794282. 18794282 PMC2583594

[3] Wang M, Krauss JL, Domon H, Hosur KB, Liang S et al. (2010) Microbial hijacking of complement-toll-like receptor crosstalk. Sci Signal 3: ra11–: ra11 PubMed: 20159852. 10.1126/scisignal.2000697 PMC2824906 20159852

[4] Harnett W, Harnett MM (2010) Helminth-derived immunomodulators: can understanding the worm produce the pill? Nat Rev Immunol 10: 278-284. doi:10.1038/nri2730. PubMed: 20224568. 20224568

[5] Kawai T, Akira S (2011) Toll-like receptors and their crosstalk with other innate receptors in infection and immunity. Immunity 34: 637-650. doi:10.1016/j.immuni.2011.05.006. PubMed: 21616434. 21616434

[6] Stremler KE, Stafforini DM, Prescott SM, Zimmerman GA, McIntyre TM (1989) An oxidized derivative of phosphatidylcholine is a substrate for the platelet-activating factor acetylhydrolase from human plasma. J Biol Chem 264: 5331-5334. PubMed: 2494162. 2494162

[7] Aiyar N, Disa J, Ao Z, Ju H, Nerurkar S et al. (2007) Lysophosphatidylcholine induces inflammatory activation of human coronary artery smooth muscle cells. Mol Cell Biochem 295: 113-120. doi:10.1007/s11010-006-9280-x. PubMed: 16896535. 16896535

[8] Kougias P, Chai H, Lin PH, Lumsden AB, Yao Q et al. (2006) Lysophosphatidylcholine and secretory phospholipase A2 in vascular disease: mediators of endothelial dysfunction and atherosclerosis. Med Sci Monit 12: RA5-16. PubMed: 16369478. 16369478

[9] Steinbrecher UP, Zhang HF, Lougheed M (1990) Role of oxidatively modified LDL in atherosclerosis. Free Radic Biol Med 9: 155-168. doi:10.1016/0891-5849(90)90728-2. PubMed: 2227530. 2227530

[10] Berliner JA, Navab M, Fogelman AM, Frank JS, Demer LL et al. (1995) Atherosclerosis: basic mechanisms. Oxidation, inflammation, and genetics. Circulation 91: 2488-2496. doi:10.1161/01.CIR.91.9.2488. PubMed: 7729036. 7729036

[11] Chisolm GM, Steinberg D (2000) The oxidative modification hypothesis of atherogenesis: an overview. Free Radic Biol Med 28: 1815-1826. doi:10.1016/S0891-5849(00)00344-0. PubMed: 10946223. 10946223

[12] Ryborg AK, Grøn B, Kragballe K (1995) Increased lysophosphatidylcholine content in lesional psoriatic skin. Br J Dermatol 133: 398-402. doi:10.1111/j.1365-2133.1995.tb02667.x. PubMed: 8546994. 8546994

[13] Mehta D, Gupta S, Gaur SN, Gangal SV, Agrawal KP (1990) Increased leukocyte phospholipase A2 activity and plasma lysophosphatidylcholine levels in asthma and rhinitis and their relationship to airway sensitivity to histamine. Am Rev Respir Dis 142: 157-161. doi:10.1164/ajrccm/142.1.157. PubMed: 2368964. 2368964

[14] George J, Harats D, Gilburd B, Levy Y, Langevitz P et al. (1999) Atherosclerosis-related markers in systemic lupus erythematosus patients: the role of humoral immunity in enhanced atherogenesis. Lupus 8: 220-226. doi:10.1191/096120399678847597. PubMed: 10342715. 10342715

[15] Stewart CR, Stuart LM, Wilkinson K, van Gils JM, Deng J et al. (2010) CD36 ligands promote sterile inflammation through assembly of a Toll-like receptor 4 and 6 heterodimer. Nat Immunol 11: 155-161. doi:10.1038/ni.1836. PubMed: 20037584. 20037584 PMC2809046

[16] Golodne DM, Monteiro RQ, Graca-Souza AV, Silva-Neto MA, Atella GC (2003) Lysophosphatidylcholine acts as an anti-hemostatic molecule in the saliva of the blood-sucking bug Rhodnius prolixus. J Biol Chem 278: 27766-27771. doi:10.1074/jbc.M212421200. PubMed: 12740385. 12740385

[17] Shamshiev AT, Ampenberger F, Ernst B, Rohrer L, Marsland BJ et al. (2007) Dyslipidemia inhibits Toll-like receptor-induced activation of CD8alpha-negative dendritic cells and protective Th1 type immunity. J Exp Med 204: 441-452. doi:10.1084/jem.20061737. PubMed: 17296788. 17296788 PMC2118729

[18] Kannan Y, Sundaram K, Aluganti Narasimhulu C, Parthasarathy S, Wewers MD (2012) Oxidatively modified low density lipoprotein (LDL) inhibits TLR2 and TLR4 cytokine responses in human monocytes but not in macrophages. J Biol Chem 287: 23479-23488. doi:10.1074/jbc.M111.320960. PubMed: 22613713. 22613713 PMC3390624

[19] Kabarowski JH (2009) G2A and LPC: regulatory functions in immunity. Prostaglandins Other Lipid Mediat 89: 73-81. doi:10.1016/j.prostaglandins.2009.04.007. PubMed: 19383550. 19383550 PMC2740801

[20] Xu Y (2002) Sphingosylphosphorylcholine and lysophosphatidylcholine: G protein-coupled receptors and receptor-mediated signal transduction. Biochim Biophys Acta 1582: 81-88. doi:10.1016/S1388-1981(02)00140-3. PubMed: 12069813. 12069813

[21] Edfeldt K, Swedenborg J, Hansson GK, Yan ZQ (2002) Expression of toll-like receptors in human atherosclerotic lesions: a possible pathway for plaque activation. Circulation 105: 1158-1161. PubMed: 11889007. 11889007

[22] Miller YI (2005) Toll-like receptors and atherosclerosis: oxidized LDL as an endogenous Toll-like receptor ligand. Future Cardiol 1: 785-792. doi:10.2217/14796678.1.6.785. PubMed: 19804052. 19804052

[23] Erridge C (2009) The roles of Toll-like receptors in atherosclerosis. J Innate Immun 1: 340-349. doi:10.1159/000191413. PubMed: 20375591. 20375591

[24] Seimon TA, Nadolski MJ, Liao X, Magallon J, Nguyen M et al. (2010) Atherogenic lipids and lipoproteins trigger CD36-TLR2-dependent apoptosis in macrophages undergoing endoplasmic reticulum stress. Cell Metab 12: 467-482. doi:10.1016/j.cmet.2010.09.010. PubMed: 21035758. 21035758 PMC2991104

[25] Magalhaes K, Almeida PE, Atella G, Maya-Monteiro CM, Castro-Faria-Neto H et al. (2010) Schistosomal-derived lysophosphatidylcholine are involved in eosinophil activation and recruitment through Toll-like receptor-2-dependent mechanisms. J Infect Dis 202: 1369-1379. 20863227 10.1086/656477

[26] Mankan AK, Lawless MW, Gray SG, Kelleher D, McManus R (2009) NF-kappaB regulation: the nuclear response. J Cell Mol Med 13: 631-643. doi:10.1111/j.1582-4934.2009.00632.x. PubMed: 19438970. 19438970 PMC3822870

[27] Lo HW, Hsu SC, Ali-Seyed M, Gunduz M, Xia W et al. (2005) Nuclear interaction of EGFR and STAT3 in the activation of the iNOS/NO pathway. Cancer Cell 7: 575-589. doi:10.1016/j.ccr.2005.05.007. PubMed: 15950906. 15950906

[28] Aktan F, Henness S, Roufogalis BD, Ammit AJ (2003) Gypenosides derived from Gynostemma pentaphyllum suppress NO synthesis in murine macrophages by inhibiting iNOS enzymatic activity and attenuating NF-kappaB-mediated iNOS protein expression. Nitric Oxide 8: 235-242. doi:10.1016/S1089-8603(03)00032-6. PubMed: 12895433. 12895433

[29] Pautz A J; Hahn S, Nowag S, Voss C et al. (2010) Regulation of the expression of inducible nitric oxide synthase. Nitric Oxide 23: 75-93. doi:10.1016/j.niox.2010.04.007. PubMed: 20438856. 10.1016/j.niox.2010.04.007 PubMed: 20438856 20438856

[30] Cole JE, Georgiou E, Monaco C (2010) The expression and functions of toll-like receptors in atherosclerosis. Mediat Inflamm 2010: 393946 PubMed: 20652007 10.1155/2010/393946 PMC2905957 20652007

[31] Cho WH, Park T, Park YY, Huh JW, Lim CM et al. (2012) Clinical significance of enzymatic lysophosphatidylcholine (LPC) assay data in patients with sepsis. Eur J Clin Microbiol Infect Dis 31: 1805-1810. doi:10.1007/s10096-011-1505-6. PubMed: 22167258. 22167258

[32] Yan JJ, Jung JS, Lee JE, Lee J, Huh SO et al. (2004) Therapeutic effects of lysophosphatidylcholine in experimental sepsis. Nat Med 10: 161-167. doi:10.1038/nm989. PubMed: 14716308. 14716308

[33] Murch O, Collin M, Sepodes B, Foster SJ, Mota-Filipe H et al. (2006) Lysophosphatidylcholine reduces the organ injury and dysfunction in rodent models of gram-negative and gram-positive shock. Br J Pharmacol 148: 769-777. PubMed: 16751791. 16751791 10.1038/sj.bjp.0706788 PMC1617069

[34] Jackson SK, Abate W, Parton J, Jones S, Harwood JL (2008) Lysophospholipid metabolism facilitates Toll-like receptor 4 membrane translocation to regulate the inflammatory response. J Leukoc Biol 84: 86-92. doi:10.1189/jlb.0907601. PubMed: 18403647. 18403647

[35] Liu Y, Shepherd EG, Nelin LD (2007) MAPK phosphatases--regulating the immune response. Nat Rev Immunol 7: 202-212. doi:10.1038/nri2035. PubMed: 17318231. 17318231

[36] Qian F, Deng J, Gantner BN, Flavell RA, Dong C et al. (2012) Map kinase phosphatase 5 protects against sepsis-induced acute lung injury. Am J Physiol Lung Cell Mol Physiol 302: L866-L874. doi:10.1152/ajplung.00277.2011. PubMed: 22307906. 22307906 PMC3362165

[37] Sato S, Nomura F, Kawai T, Takeuchi O, Mühlradt PF et al. (2000) Synergy and cross-tolerance between toll-like receptor (TLR) 2- and TLR4-mediated signaling pathways. J Immunol 165: 7096-7101. PubMed: 11120839. 11120839 10.4049/jimmunol.165.12.7096

[38] Guha M, Mackman N (2001) LPS induction of gene expression in human monocytes. Cell Signal 13: 85-94. doi:10.1016/S0898-6568(00)00149-2. PubMed: 11257452. 11257452

[39] Jonassen JA, Kohjimoto Y, Scheid CR, Schmidt M (2005) Oxalate toxicity in renal cells. Urol Res 33: 329-339. doi:10.1007/s00240-005-0485-3. PubMed: 16284883. 16284883

[40] Bzowska M, Nogieć A, Skrzeczyńska-Moncznik J, Mickowska B, Guzik K et al. (2012) Oxidized LDLs inhibit TLR-induced IL-10 production by monocytes: a new aspect of pathogen-accelerated atherosclerosis. Inflammation 35: 1567-1584. doi:10.1007/s10753-012-9472-3. PubMed: 22556042. 22556042 PMC3397235

[41] Huang A, Li C, Kao RL, Stone WL (1999) Lipid hydroperoxides inhibit nitric oxide production in RAW264.7 macrophages. Free Radic Biol Med 26: 526-537. doi:10.1016/S0891-5849(98)00236-6. PubMed: 10218641. 10218641

[42] Schmid W, Lee A, Son J, Koller E, Volf I (2008) Hypochlorite-oxidized low density lipoproteins reduce production and bioavailability of nitric oxide in RAW 264.7 macrophages by distinct mechanisms. Life Sci 83: 50-58. doi:10.1016/j.lfs.2008.05.002. PubMed: 18558412. 18558412

[43] Silva-Neto MA, Carneiro AB, Silva-Cardoso L, Atella GC (2012) Lysophosphatidylcholine (2012): A Novel Modulator of Trypanosoma cruzi Transmission. J Parasitol Res, 2012: 625838 PubMed: 22132309 10.1155/2012/625838 PMC3206328 22132309

[44] McDonald D, Carrero G, Andrin C, de Vries G, Hendzel MJ (2006) Nucleoplasmic beta-actin exists in a dynamic equilibrium between low-mobility polymeric species and rapidly diffusing populations. J Cell Biol 172: 541-552. doi:10.1083/jcb.200507101. PubMed: 16476775. 16476775 PMC2063674

[45] Underhill DM, Ozinsky A, Hajjar AM, Stevens A, Wilson CB et al. (1999) The Toll-like receptor 2 is recruited to macrophage phagosomes and discriminates between pathogens. Nature 401: 811-815. doi:10.1038/44605. PubMed: 10548109. 10548109

[46] Akashi S, Shimazu R, Ogata H, Nagai Y, Takeda K et al. (2000) Cutting edge: cell surface expression and lipopolysaccharide signaling via the toll-like receptor 4-MD-2 complex on mouse peritoneal macrophages. J Immunol 164: 3471-3475. PubMed: 10725698. 10725698 10.4049/jimmunol.164.7.3471

[47] Mizushima S, Nagata S (1990) pEF-BOS, a powerful mammalian expression vector. Nucleic Acids Res 18: 5322. doi:10.1093/nar/18.17.5322. PubMed: 1698283. 1698283 PMC332193

[48] Hajjar AM, O’Mahony DS, Ozinsky A, Underhill DM, Aderem A et al. (2001) Cutting edge: functional interactions between toll-like receptor (TLR) 2 and TLR1 or TLR6 in response to phenol-soluble modulin. J Immunol 166: 15-19. PubMed: 11123271. 11123271 10.4049/jimmunol.166.1.15

[49] Schindler U, Baichwal VR (1994) Three NF-kappa B binding sites in the human E-selectin gene required for maximal tumor necrosis factor alpha-induced expression. Mol Cell Biol 14: 5820-5831. doi:10.1128/MCB.14.9.5820. PubMed: 7520526. 7520526 PMC359108

[50] Sweetser MT, Hoey T, Sun YL, Weaver WM, Price GA et al. (1998) The roles of nuclear factor of activated T cells and ying-yang 1 in activation-induced expression of the interferon-gamma promoter in T cells. J Biol Chem 273: 34775-34783. doi:10.1074/jbc.273.52.34775. PubMed: 9857002. 9857002

[51] Stuart LM, Deng J, Silver JM, Takahashi K, Tseng AA et al. (2005) Response to Staphylococcus aureus requires CD36-mediated phagocytosis triggered by the COOH-terminal cytoplasmic domain. J Cell Biol 170: 477-485. doi:10.1083/jcb.200501113. PubMed: 16061696. 16061696 PMC2171464

[52] Miller I, Radwan M, Strobl B, Müller M, Gemeiner M (2006) Contribution of cell culture additives to the two-dimensional protein patterns of mouse macrophages. Electrophoresis 27: 1626-1629. doi:10.1002/elps.200500744. PubMed: 16532519. 16532519

